# Hydatidose multiple à localisation inhabituelle, pancréatique et pelvienne : à propos d’un cas

**DOI:** 10.11604/pamj.2017.26.214.11806

**Published:** 2017-04-24

**Authors:** Lamyae Amro, Saloua El Fadili, Hind Serhane, Hafsa Sajiai, Salma Ait Batahar

**Affiliations:** 1 Service de Pneumologie, Hôpital Arrazi, CHU Mohamed VI, Laboratoire PCIM, UCAM, Marrakech, Maroc

**Keywords:** Hydatique cyst, unusual localization, pancreas, pelvis, Hydatique cyst, unusual localization, pancreas, pelvis

## Abstract

Le kyste hydatique est une pathologie infectieuse assez fréquente au Maroc. Les localisations pelvienne et pancréatique de cette parasitose sont rares voir exceptionnelles. Nous rapportons l'observation d'une patiente de 66 ans, opérée il y a 6 ans pour kyste hydatique hépatique, qui s'est présenté pour douleurs thoraciques avec hydatidoptysie. La radiographie thoracique a objectivé un hydropneumothorax gauche. La TDM thoraco-abdomino-pelvienne a mis en évidence une formation liquidienne médiastinale ainsi que des multiples lésions kystiques hépatiques, pancréatique (isthme), sous diaphragmatique gauche et pelvienne. La sérologie hydatique était positive. Le traitement a consisté à une thoracotomie associé au traitement médical.

## Abstract (en)

Hydatid cyst is an infectious disease which is quite frequent in Morocco. Pelvic and pancreatic locations of this parasitic infection are rare, namely exceptional. We report the case of a 66-year old patient who was operated for liver hydatic cyst 6 years before, presenting for chest pain associated with hydatidoptysis. Chest X-ray objectified left hydropneumothorax. Thoraco-abdominopelvic CT showed mediastinal liquid mass as well as multiple hepatic, pancreatic (isthmus), pelvic and left under diaphragmatic cystic lesions. Hydatid serology was positive. Treatment involved thoracotomy associated with medical treatment.

## Introduction

L'hydatidose est une anthropozoonose due au développement, chez l'homme, de la forme larvaire du taenia Echinococcus Granulosis. Elle est répandue de façon endémique en Afrique du Nord et dans certains pays du pourtour du bassin méditerranéen, en Nouvelle-Zélande, en Australie et en Amérique où elle représente un véritable problème de Santé publique. La prévalence de l'hydatidose est très variable. Le Maghreb est une zone intermédiaire [1]. Le Maroc, pays d'élevage traditionnel, se place parmi les pays les plus infestés par cette parasitose. Outre les localisations hépatiques et pulmonaires, qui sont les plus fréquentes, l'hydatidose peut se développer dans n'importe quel organe [2]. Les kystes hydatiques à localisation pelvienne et pancréatique font partie de ces cas rares et trompeurs. Ils représentant moins de 1% de l'ensemble des localisations. Le diagnostic, qui repose sur la radiologie et la sérologie, devient difficile en cas de localisation atypique en raison du diagnostic différentiel avec d'autres lésions kystiques. Nous rapportons un cas d'une hydatidose multiple pulmonaire, hépatique, pancréatique et pelvienne.

## Patient et observation

**Figure 1:**
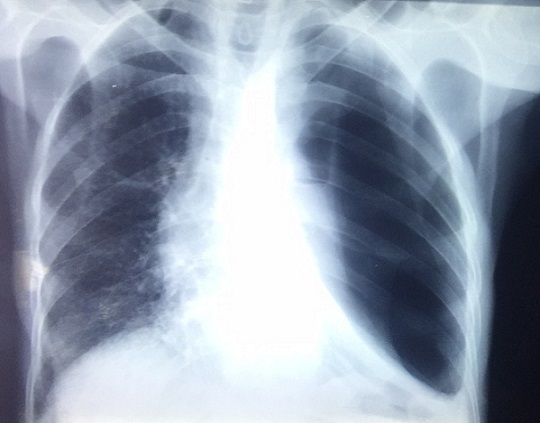
Radiographie thoracique montre une une hyperclarté de tout l’hémithorax gauche avec présence de deux images hydroaériques et une réaction pleurale avec refoulement des éléments du médiastin

**Figure 2:**
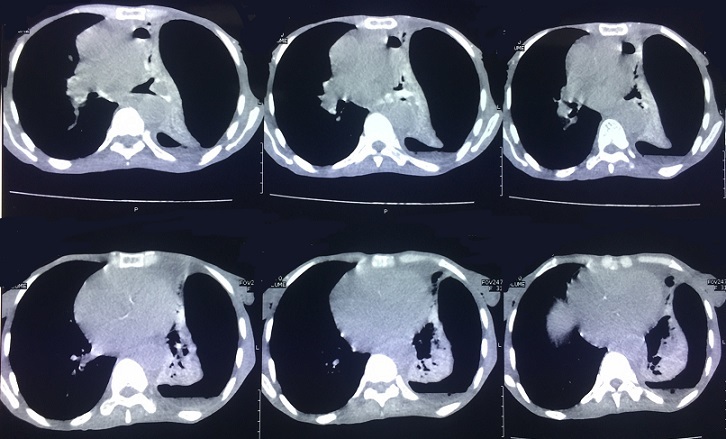
TDM thoracique a mis en évidence hydropneumothorax gauche de grande abondance semblant communiquer avec une image hydroaérique au niveau du moignon pulmonaire collabé associé à une formation liquidienne se projetant à hauteur du médiastin pouvant être en rapport avec un kyste hydatique

**Figure 3:**
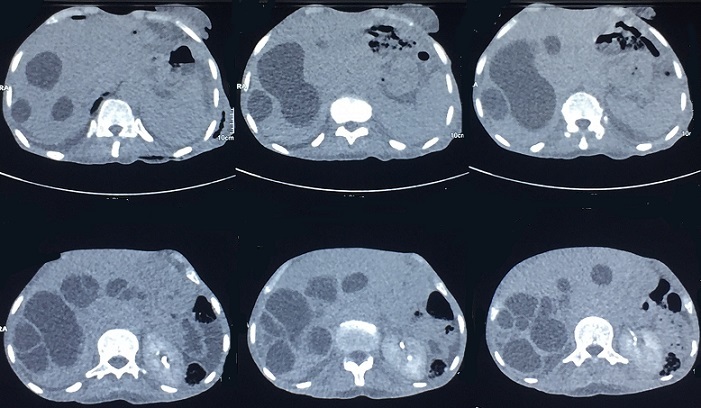
TDM abdominale objective de multiples lésions kystiques hépatiques, pancréatique (isthme) et sous diaphragmatique

**Figure 4:**
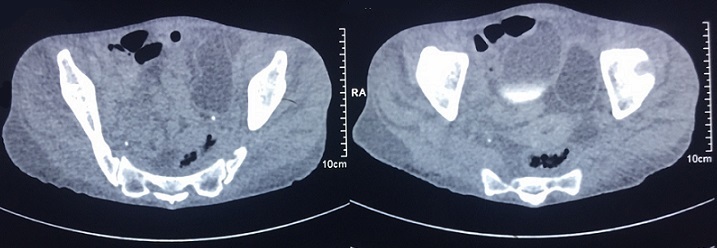
TDM pelvienne montre une formation kystique pelvienne latéro-vésicale gauche

Patiente âgée de 66ans, femme au foyer, résidante en milieu rural et ayant un contact avec les chiens. Elle a été opérée pour kyste hydatique du foie il y a 3 ans sans autres antécédents pathologiques. La symptomatologie clinique remonte à 3 ans par l'installation progressive d'un syndrome bronchique fait de toux ramenant des expectorations jaunâtres avec un épisode d'hydatidoptysie associé à des douleurs thoraciques gauches en point de coté et une dyspnée, le tout évoluant dans un contexte de sensations fébriles, sueurs nocturnes et fléchissement de l'état général. L'examen physique a objectivé un syndrome d'épanchement mixte de l'hémithorax gauche et un ictère cutanéomuqueux généralisé. La radiographie du thorax a montré une hyperclarté de tout l'hémithorax gauche avec présence de deux images hydroaériques au niveau du moignon pulmonaire, une réaction pleurale avec refoulement des éléments du médiastin (Figure 1). La TDM thoracique a mis en évidence un hydropneumothorax gauche de grande abondance semblant communiquer avec une image hydroaérique au niveau du moignon pulmonaire associé à une formation liquidienne se projetant à hauteur du médiastin pouvant être en rapport avec un kyste hydatique (Figure 2). La recherche de scolex dans le liquide pleural était négative mais la sérologie hydatique était positive. La TDM abdomino-pelvienne a objectivé de multiples lésions kystiques hépatiques, pancréatique (isthme) et pelvienne en faveur de kystes hydatiques (Figure 3, Figure 4). Le traitement a consisté à une thoracotomie associé au traitement médical. Cependant la patiente est décédée avant l'intervention chirurgicale.

## Discussion

Le kyste hydatique est généralement localisé au niveau hépatique et pulmonaire. La localisation pancréatique est rare, elle représente 0,2 à 2 % [3]. L'infestation du pancréas se fait par diffusion hématogène, par invasion péri-pancréatique, ou par l'extension locale à partir du foie à travers les canaux pancréatobiliaires [4]. La localisation pancréatique est isolée dans 91 % des cas avec une légère prédilection pour la portion céphalique (57% des cas). L'atteinte corporéale et caudale se voient, respectivement, dans 24 et 19 % des cas. La localisation est périphérique dans les deux tiers des cas [5]. La taille du kyste est variable, pouvant aller de quelques millimètres à plus de 20 cm [6]. Dans notre cas, la localisation était isthmique de 9 mm de diamètre. La symptomatologie, souvent insidieuse après une longue évolution, dépend du siège du kyste. Les douleurs abdominales de l'étage sus-ombilical constituent le motif de consultation le plus fréquent. Bedioui et al a rapporté 3 cas de kyste hydatique primitif du pancréas révélé par des douleurs abdominales [6]. Certaines complications évolutives peuvent être révélatrices du kyste hydatique du pancréas telles qu'un ictère rétentionel en cas de kystes céphaliques, une suppuration du kyste, une pancréatite chronique, une rupture intra-ou rétropéritonéale du kyste, l'ouverture du kyste dans les organes de voisinage et l'hypertension portale segmentaire. Un infarctus mésentérique a été décrit comme la conséquence d'une thrombose de l'artère mésentérique supérieure en rapport avec une compression hydatique [6]. La fistulisation du kyste dans le Wirsung peut être responsable de poussées de pancréatite aigue récidivantes, voire de wirsungorragie [6]. Les cas de kyste hydatique du pancréas rapporté par Chammakhi-Jemli et al, et Pouget et al, étaient révélés par une pancréatite aigue [3, 7]. L´échographie, la tomodensitométrie et l´imagerie par résonance magnétique nucléaire, reconnaissent sans difficulté la lésion kystique pancréatique, mais la difficulté est de rattacher cette lésion à la maladie hydatique [5]. Les caractéristiques radiologiques qui permettent de distinguer les kystes hydatiques des autres lésions kystiques du pancréas sont la présence de calcifications curvilignes de la paroi kystique. Autres éléments d'orientation sont: la présence de vésicules filles, de débris connus sous le nom de sable hydatique, de cloisons, ou de la membrane proligère décollée. En dépit de ces caractéristiques assez spécifiques, les kystes hydatiques avec des localisations inhabituelles (telles que le pancréas) présentent un véritable défi diagnostique [4]. Une sérologie hydatique positive affirme habituellement la maladie hydatique, mais le pourcentage de positivité semble plus faible par rapport à l'hydatidose hépatique et pulmonaire [7]. La négativité de la sérologie n'élimine pas la nature hydatique d'une masse kystique pancréatique. Ainsi, la confrontation des données épidémiologiques, cliniques, radiologiques (échographie, tomodensitométrie, IRM et éventuellement, échoendoscopie) et immunologiques permet, dans la majorité des cas, de confirmer la nature hydatique d'une masse kystique pancréatique [6]. Le traitement du kyste hydatique du pancréas est chirurgical. Il dépend du siège du kyste et de l'existence d'une éventuelle fistule pancréatique. Concernant les kystes céphaliques, le traitement consiste en une résection du dôme saillant avec drainage de la cavité résiduelle associée. Pour la localisation corporéocaudal du kyste, une pancréatectomie gauche permettra d'emporter le kyste et de suturer le pancréas en tissu sain. Le traitement médical adjuvant par l'albendazole est indiqué en cas de rupture peropératoire du kyste ou d'hydatidose multiple [4].

L´hydatidose pelvienne est rare. Son incidence est comprise entre 0,30 et 4,27% des localisations hydatiques, dont 80 % des cas implique la sphère génitale [2]. La majorité des cas rapportés dans la littérature concerne des patientes âgées entre 20 et 40 ans [2], notre patiente était âgée de 66 ans. L'hydatidose pelvienne est exceptionnelle avant l'âge de 10 ans, 7 cas de kyste hydatique rétrovésical ont été raportés par Hafsa [8] et Ben Ahmed [9]. Un double mécanisme étiopathogénique serait probablement incriminé: d'une part, la greffe hématogène primitive d'embryons hexacanthes comme dans le cas du Kyste hydatique rétropéritonéal, d'autre part, la greffe secondaire dans le cul-de sac de Douglas de protoscolex provenant de la fissuration de kystes hydatiques abdominaux. Dans notre série il s'agit très probablement de la deuxième théorie puisque la patiente a été opérée pour un Kyste hydatique du foie 3 ans auparavant. Une autre voie exceptionnelle peut expliquer la localisation rétrovésicale d'un Kyste hydatique, il s'agit de la voie lymphatique par emprunt du système veineux de Retzius et les anastomoses de Schmiedel [9]. L'anamnèse joue un rôle capital dans le diagnostic de l'hydatidose en recherchant les facteurs de risque tel que le contact avec les chiens et le contexte socioprofessionnel. L'interrogatoire permet aussi de rechercher la notion d'intervention antérieure sur une hydatidose hépatique et l'existence d'un accident aigu évoquant la rupture ou la fissuration d'un kyste hydatique [10].

Cette affection a une évolution lente et silencieuse ce qui explique l'apparition tardive des signes cliniques. Pour la localisation pelvienne, les signes d'irritation vésicale sont les motifs les plus fréquents de consultation. Il peut s'agir d'une masse hypogastrique palpable (29 %), de douleurs pelviennes (24 %), de rétention urinaire (14%) ou d'hydaturie (9,5%). Parfois, il peut s'agir d'une rétention aiguë d'urines, de troubles mictionnels (dysurie, pollakiurie), de troubles du transit ou de lombalgies secondaires à une compression urétérale obstructive [9]. L'apparition d'une hydaturie est un signe pathognomonique de fissuration du kyste dans la vessie [9]. Parfois, le kyste est découvert à l'occasion d'une complication: suppuration du kyste, état de choc après sa rupture ou insuffisance rénale par compression urétérale [9]. Dans la série de Laghzaoui Boukaidi et al, Le kyste hydatique a été révélé dans six cas par une masse pelvienne ou abdominopelvienne associée à des algies sans caractère particulier, dans un cas le kyste hydatique a été découvert de façon fortuite au cours d'une échographie réalisée pour des métrorragies sur une grossesse de 32 semaines d'aménorrhée. Chez un autre cas, le kyste hydatique a été découvert lors de douleurs pelviennes isolées, deux patientes avaient une stérilité primaire, les métrorragies étaient notés dans un cas de même que les troubles du transit [10]. Il n'y a aucun examen sérologique ou immunologique pathognomonique de la maladie hydatique. Actuellement, l'échographie et la tomodensitométrie sont réalisées dans le cadre du diagnostic positif et topographique de la maladie hydatique avec une spécificité et une sensibilité élevées. Ces 2 examens montrent l'aspect typique d'un kyste hydatique qui peut être uni- ou multivésiculaire [11]. L'imagerie par résonance magnétique n'est pas une technique de première intention dans la maladie hydatique. Elle ne trouve sa justification que lorsque les autres imageries ne permettent pas d'établir un diagnostic certain [9]. Le kyste se présente comme une masse circonscrite, en hyposignal en séquence pondérée T1, en hypersignal T2 et qui se modifie peu ou pas après injection de produit de contraste. La mise en évidence de vésicules filles avec des septa en hyposignal T1 et T2 est pathognomonique de kyste hydatique. Si le kyste est compliqué, il présente un signal hétérogène aussi bien en pondération T1 que T2 avec un rehaussement de la paroi après injection intraveineuse de produit de contraste [9]. Le diagnostic différentiel se fait avec toutes les tumeurs kystiques ou mixtes rétro péritonéales (kystes dermoïdes), les abcès à pyogènes ou les abcès tuberculeux, les kystes de l'ovaire, les hydrosalpinx, les tumeurs ovariennes, les fibromes utérins surtout sous séreux [2]. La chirurgie est le traitement de choix de l'hydatidose pelvienne. La kystectomie totale est le procédé idéal, mais la kystectomie partielle ou subtotale peut être réalisée pour éviter de blesser les organes de voisinage. Les agents scolocides les plus utilisés sont le NaCl ou l'eau oxygénée. L'exploration doit rechercher d'autres localisations hydatiques qui seront traitées en même temps. Le mébendazole ou l'albendazole sont utilisés en traitement adjuvant de la chirurgie pour minimiser les récidives. Un recul de 2 ans est nécessaire pour juger de l'efficacité du traitement [11].

## Conclusion

L'hydatidose pancréatique et pelvienne sont des localisations rare de l'échinococcose. L'association des deux est encore plus rare voir exceptionnelle. Le diagnostic positif repose sur les données épidémiologiques, cliniques, biologiques et surtout radiologiques. Cependant devant de ces localisations atypiques, il devient moins évident vu le problème de diagnostic différentiel avec d'autres lésions kystique bien plus fréquentes. Le traitement est essentiellement chirurgical parfois médical. La prévention reste le meilleur moyen pour diminuer l'incidence de cette pathologie.

## References

[1] Venara Aurélien, Mehinto Delphin K, Lermite Émilie, Chabasse Dominique, Hamy Antoine, Arnaud Jean-Pierre (2011). Localisations primitives inhabituelles du kyste hydatique. Presse Med.

[2] Elfazazi H, Kouach J, Babahabib A, Oukabli M, Hafidi MR, Salek G, Moussaoui RD, Dehayni M (2010). Kyste hydatique primitif pelvien. Imagerie de la Femme.

[3] Pouget Y, Mucci S, O'Toole D, Lermite E, Aubé C, Hamy A (2009). Pancréatite aiguë récidivante révélant un kyste hydatique du pancreas. Rev Med Interne.

[4] Elmadi A, Khattala K, Elbouazzaoui A, Rami M, Labib I, Harandou M, Afifi A, Bouabdallah Y (2010). Journal de Pédiatrie et de Puériculture.

[5] Fadil A, Ait Bolbarod A, El Fares F (2000). Kyste hydatique du pancréas : À propos d?une observation. Ann chir.

[6] Bedioui H, Chebbi F, Ayadi S, Daghfous A, Bakhtri M, Jouini M, Ftériche F, Ksantini R, Ksantini R, Kacem M, Safta ZB (2008). Kyste hydatique primitif du pancréas : diagnostic et modalités chirurgicales : à propos de trois cas. Gastroenterol Clin Biol.

[7] Chammakhi-Jemli C, Mekaouer S, Miaoui A, Daghfous A, Mzabi H, Cherif A, Daghfous MH (2010). Pancréatite aiguë révélatrice d?un kyste hydatique du pancréas. J Radiol.

[8] Hafsa C, Golli M, Kriaa S, Salem R, Jerbi Omezzine S, Bourogaa S, Belguith M, Nouri A, Gannouni A (2007). Le kyste hydatique rétrovésical chez l'enfant : à propos de trois cas. J Radiol.

[9] Ben Ahmed Y, Khemekhem R, Nouira F, Boukedi A, Rahay H, Charieg A, Ghorbel S, Jlidi S, Chaouachi B (2012). Kyste hydatique retrovésical chez l'enfant : à propos de quatre cas. Journal de pédiatrie et de puériculture.

[10] Laghzaoui Boukaid M, Bouhya S, Soummani A, Hermas S, Bennan O, Sefrioui O, Aderdou M (2001). Kystes hydatiques pelviens : à propos de huit cas. Gynécol Obstét Fertil.

[11] Tajdine MT, Daali M (2007). Kyste hydatique pelvien isolé: à propos de 1 cas pédiatrique. Archives de pédiatrie.

